# Photobiomodulation with or without ILIB in diabetic peripheral neuropathy: a randomized pilot trial

**DOI:** 10.1007/s10103-026-04948-8

**Published:** 2026-07-10

**Authors:** Joelita de Alencar Fonseca Santos, José Erivelton de Souza Maciel Ferreira, João Wesley da Silva Galvão, Ainoã de Oliveira Lima, Elaine Cristina Sá de Almeida, Lukénia André Lukelo, Gyrlany Alves Pereira, Thiago Moura de Araújo

**Affiliations:** Postgraduate Program in Nursing, University for International Integration of the Afro-Brazilian Lusophony, Redenção, Brazil

**Keywords:** Diabetic Neuropathies, Photobiomodulation Therapy, Low-Level Light Therapy, Pain Measurement, Peripheral Neuropathy

## Abstract

Diabetic peripheral neuropathy is a disabling chronic complication of diabetes mellitus, associated with neuropathic pain, sensory dysfunction, and reduced quality of life. Because pharmacological treatments often provide only partial relief, photobiomodulation therapy (PBMT) has emerged as a promising non-pharmacological strategy with metabolic, anti-inflammatory, and neuromodulatory effects. To assess the feasibility of local PBMT, administered alone or combined with intravascular laser irradiation of blood (ILIB), and to explore preliminary short-term changes in neuropathic pain, neuropathic symptoms, and sensory responses in individuals with diabetic peripheral neuropathy. This prospective randomized controlled pilot trial included 26 participants allocated to three groups: local PBMT, local PBMT combined with ILIB, and sham control. All randomized participants completed 12 sessions over 23 days. Neuropathic pain intensity was assessed using the Visual Analog Scale, and neuropathic symptoms were evaluated using the Michigan Neuropathy Screening Instrument. Friedman and McNemar tests were used for exploratory within-group and paired analyses, and a linear mixed-effects model evaluated pain trajectory over time. Neuropathic pain intensity decreased across the five assessment points (p < 0.001), with significant intragroup reduction in the local PBMT group (p = 0.007). Improvements were observed in selected symptoms, including numbness, cramps, and dry skin with fissures. The mixed-effects model showed a significant effect of time on pain reduction (beta = -0.78; p = 0.001), although no significant group x time interaction was detected. Local PBMT, administered alone or combined with ILIB, was feasible in this outpatient pilot trial and was followed by preliminary short-term reductions in neuropathic pain and selected neuropathic symptoms. However, no definitive between-group superiority was demonstrated, and the findings should be interpreted cautiously because of the pilot design, small sample size, heterogeneity in baseline pain intensity, potential placebo effects, and short follow-up period.

## Introduction

Diabetic peripheral neuropathy (DPN) is one of the most prevalent and disabling chronic complications of diabetes mellitus, affecting approximately 6% to 34% of individuals with the disease [1]. The most common presentation is distal symmetric polyneuropathy, characterized by progressive involvement of sensory and autonomic nerve fibers, with manifestations including burning pain, paresthesia, loss of protective sensation, thermal alterations, and proprioceptive dysfunction [2]. Neuropathic pain associated with this complication substantially reduces quality of life, interferes with daily activities, and contributes to an increased risk of ulceration and amputation [3].

From a pathophysiological perspective, DPN results from a complex interaction among chronic hyperglycemia, dyslipidemia, oxidative stress, low-grade inflammation, and endoneurial microvascular impairment ([2]; [4]). Damage affects small fibers responsible for nociception and sudomotor regulation, as well as larger fibers associated with vibratory sensation and postural control [4]. Neuropathy progression has also been linked to mitochondrial dysfunction and reduced neural perfusion, phenomena that sustain pain persistence and sensory abnormalities [5].

Although glycemic control remains the central therapeutic target, no intervention has consistently demonstrated the ability to fully reverse neuropathic progression [4, 6]. Symptomatic treatment of painful DPN is based predominantly on anticonvulsants, antidepressants, opioids, and topical agents; however, these approaches are frequently limited by adverse effects, incomplete response, and long-term tolerability concerns [1, 7]. Given these limitations, non-pharmacological interventions with potential metabolic, anti-inflammatory, and neuromodulatory effects have been investigated, including photobiomodulation therapy (PBMT).

PBMT delivered through low-level red or infrared laser sources may interact with mitochondrial cytochrome c oxidase, promoting adenosine triphosphate production, modulation of cellular redox status, and reduction of inflammatory mediators [8]. Evidence from peripheral nerve lesion studies suggests that PBMT may favor neuronal repair, improve nerve conduction, and reduce neuropathic pain [9]. In individuals with DPN, clinical studies and reviews have reported improvements in neuropathic pain and possible effects on nerve function, although methodological heterogeneity and limited comparative designs remain important concerns [10, 11].

In addition to local application, intravascular laser irradiation of blood (ILIB) has been proposed as a complementary systemic PBMT approach. ILIB has been associated with antioxidant, hemorheological, vasodilatory, and anti-inflammatory effects that may influence microcirculation and systemic oxidative stress [12, 13]. A recent narrative review of randomized trials suggested that ILIB may reduce pain and improve quality of life in different clinical conditions, while evidence in diabetic neuropathy remains limited and requires more rigorous comparative assessment [14]. Considering that DPN involves both local nerve injury and systemic metabolic-inflammatory mechanisms, the combination of local PBMT and ILIB has biological plausibility, but its additive clinical value remains uncertain.

Previous randomized studies have suggested potential benefits of PBMT-related approaches in DPN, but relevant gaps remain. Anju et al., [15] evaluated PBMT using a sham-controlled design and reported improvements in neuropathic pain, quality of life, and neuronal biomarkers; however, the intervention focused on local PBMT and did not examine the additive role of ILIB. Other studies and reviews have focused mainly on pain, quality of life, nerve conduction, or plantar pressure distribution, with limited assessment of longitudinal pain trajectories, specific neuropathic symptoms, and feasibility in outpatient primary care settings [10, 11, 16]. Therefore, evidence remains limited regarding whether combining local PBMT with a systemic PBMT approach such as ILIB provides an additional clinical signal beyond local treatment alone or sham procedures.

In this context, the present randomized pilot study aimed to assess the feasibility of local PBMT, administered alone or combined with ILIB, and to explore preliminary short-term changes in neuropathic pain intensity, neuropathic symptoms, and sensory responses in individuals with DPN. The study was not designed or powered to establish definitive comparative efficacy between interventions.

## Methods

### Study design

This prospective, randomized, controlled, three-arm parallel pilot trial (1:1:1 allocation ratio) was conducted between July and October 2022 at an Integrated Health Center of a public Brazilian university. The study was registered in the Brazilian Registry of Clinical Trials before the beginning of data collection, with the registration number concealed during peer review. As a pilot trial, its main objective was to assess the feasibility of the intervention protocol and to generate preliminary estimates of changes in neuropathic pain, neuropathy-related symptoms, and sensory responses following local PBMT, applied either alone or in combination with ILIB, in individuals with DPN.

The manuscript was revised according to recommendations for transparent reporting of randomized pilot trials, emphasizing feasibility, preliminary estimates, and uncertainty rather than definitive efficacy claims.

### Participants

Individuals with a diagnosis of type 1 or type 2 diabetes mellitus, aged 30 to 70 years, and with a clinical presentation consistent with symptomatic DPN were considered eligible. Symptomatic DPN was defined by neuropathic complaints in the lower limbs, including burning pain, paresthesia, pain symptoms, and/or sensory alterations. All randomized participants met this clinical criterion. Baseline VAS was used to quantify current pain intensity among eligible participants rather than as a separate severity-based exclusion criterion.

Participants were excluded if they presented active foot ulceration, acute metabolic decompensation, severe hypoglycemia, ketoacidosis, decompensated chronic cardiovascular disease, non-diabetic neuropathies, thyroid dysfunction, pregnancy, metallic implants, neoplasms, substance abuse, major mobility limitation, or major lower-limb amputation preventing protocol application. A previous history of minor amputation, such as toe or partial forefoot amputation, was not considered an exclusion criterion when the participant was clinically stable, able to attend the intervention sessions, and had remaining anatomical sites suitable for PBMT and sensory assessment. No participant had major bilateral lower-limb loss.

Participants were instructed to maintain their usual diabetes and pain-related medications during the 23-day protocol. The research team did not initiate, discontinue, or modify pharmacological therapy. Because medication regimens were not controlled by the study protocol, concurrent antidiabetic and analgesic/neuropathic pain medications were considered potential sources of residual confounding.

Initially, 32 individuals were assessed for eligibility. Six were not randomized because they were unable to attend the scheduled treatment sessions owing to transportation difficulties and access barriers to the health service. Thus, 26 participants were randomized and included in the analysis.

### Randomization, allocation concealment and blinding

Participants were randomly allocated to one of three study groups using a simple randomization procedure with a 1:1:1 allocation ratio. The allocation sequence was generated before recruitment using a computer-generated random-number list in Microsoft Excel by a researcher not involved in participant recruitment, intervention delivery, outcome assessment, or statistical analysis. Group assignments were placed in opaque, sealed, sequentially numbered envelopes.

After eligibility confirmation, written informed consent, and baseline assessment, the next envelope in the sequence was opened, and the assigned intervention was communicated to the therapist responsible for treatment delivery. This procedure was intended to preserve allocation concealment until enrollment and baseline assessment had been completed.

Because of the physical characteristics of the interventions, therapists could not be blinded. Outcome assessors were also not blinded because clinical assessments were performed by the same therapists who delivered the interventions. Participants, including those allocated to the sham-control group, were blinded to treatment allocation. The statistician remained blinded to group allocation during the primary modeling procedures. No blocking or stratification procedures were applied because this was an exploratory pilot trial.

### Intervention protocol

All participants underwent 12 treatment sessions administered at 48-hour intervals, totaling 23 days of follow-up. Interventions were delivered by trained therapists according to standardized procedures specific to each study group. The parameters were selected based on the therapeutic configuration available in the DMC Therapy EC device and on previous PBMT protocols used for neuropathic pain and DPN, while recognizing that dose-response relationships in DPN remain insufficiently established. The intervention parameters are summarized in Table 1.

**Table 1:** Intervention protocol and technical parameters

Parameter	Local PBMT	Local PBMT + ILIB	Sham control
Wavelength	660 nm red laser	660 nm red laser for local PBMT + 660 nm red laser for ILIB	Laser emission blocked
Output power	100 mW (manufacturer-reported)	100 mW local PBMT + 100 mW ILIB (manufacturer-reported)	No therapeutic light delivered
Emission mode	Continuous	Continuous	Same device handling; emission blocked
Local dose/fluence	6 J/cm² (manufacturer/programmed setting)	6 J/cm² for local PBMT	Not applicable
Beam area/spot size	0.06 cm² (manufacturer-reported)	0.06 cm² for local PBMT; ILIB spot size not independently measured and applied according to manufacturer-specific applicator configuration	Not applicable
Local irradiation time	13 s per point	13 s per point + 20 min ILIB	Same duration and positioning as active groups
Local application sites	Plantar regions of the 1st, 3rd, and 5th metatarsals; distal phalanges of the 1st and 3rd toes; dorsal region between the 1st and 2nd toes	Same local application sites	Same local positioning with blocked emission
ILIB application site	Not applicable	Transcutaneous application over the radial artery, with applicator fixed over the wrist/radial artery region	Same positioning with blocked emission when applicable
Number of sessions	12	12	12
Interval between sessions	48 h	48 h	48 h
Follow-up period	23 days	23 days	23 days
ILIB irradiation time	Not applicable	20 min	Same duration with blocked emission when applicable

PBMT, photobiomodulation therapy; ILIB, intravascular laser irradiation of blood. Device parameters were based on manufacturer-reported specifications and programmed settings. Independent optical calibration was not performed. For the ILIB procedure, spot size and fluence were not independently measured and should be interpreted according to the manufacturer-specific applicator configuration

Source: authors (2026)

The equipment used was a DMC Therapy EC photobiomodulation device (DMC, Brazil), composed of red and infrared laser sources. The present protocol used the red wavelength at 660 nm in continuous mode. Device parameters were based on manufacturer-reported settings. Independent optical power calibration was not performed during the trial.

Participants allocated to the local PBMT group received treatment using a 660-nm red laser operating in continuous mode. Laser application was performed at predefined anatomical sites on both feet, including the plantar regions of the first, third, and fifth metatarsals, the distal phalanges of the first and third toes, and the dorsal region between the first and second toes corresponding to the course of the dorsalis pedis artery.

Participants allocated to the local PBMT + ILIB group received the same local PBMT protocol applied to the feet, combined with transcutaneous ILIB. ILIB was administered over the radial artery using a 660-nm red laser in continuous mode for 20 min, with the applicator fixed over the wrist/radial artery region.

Participants allocated to the sham-control group underwent the same positioning procedures, treatment duration, therapist interaction, and device handling as those in the active intervention groups. To maintain participant blinding, laser emission was blocked by covering the laser aperture with opaque material, preventing the delivery of therapeutic light while preserving the appearance and operation of the device.

**Fig. 1:**
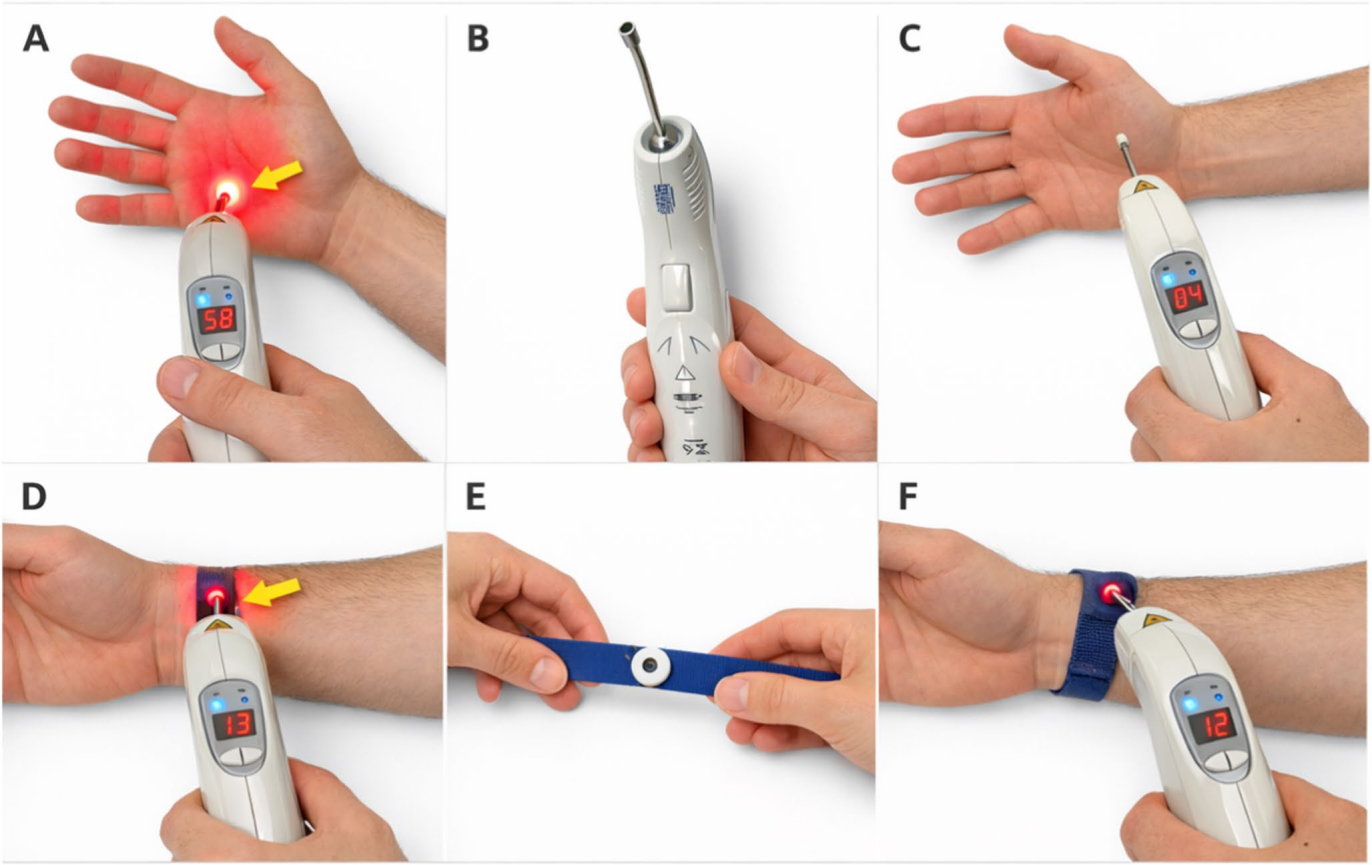
Study interventions and photobiomodulation procedures. (**A**) Local PBMT applied to predefined plantar foot sites; (**B**) DMC Therapy EC device used in the trial; (**C**) sham intervention with identical positioning and device handling procedures; (**D**) transcutaneous ILIB applied over the radial artery region; (**E**) fixation strap used for ILIB application; and (**F**) ILIB administration with the applicator secured at the wrist. Note: PBMT, photobiomodulation therapy; ILIB, intravascular laser irradiation of blood. Source: authors (2026)

Figure 1 presents the photobiomodulation device and the positioning procedures used during local PBMT, transcutaneous ILIB, and sham interventions.

### Outcomes

The primary clinical outcome was neuropathic pain intensity, assessed using the Visual Analog Scale (VAS). Secondary outcomes included neuropathic symptoms assessed using the Michigan Neuropathy Screening Instrument (MNSI) [17], namely numbness, burning pain, stabbing pain in the legs or feet, leg and/or foot cramps, muscle weakness, xeroderma, sensitivity during walking, and worsening of symptoms during the day or at night. Thermal sensitivity tests using warm and cold tubes, tactile sensitivity assessment using the 10 g monofilament, and pain sensitivity assessment using a blunt stick/needle were also performed. Assessments were conducted at baseline and repeated at five time points, on days 1, 7, 13, 19, and 23.

VAS pain intensity and MNSI symptoms were analyzed at the participant level. Sensory tests were recorded by foot and anatomical site. Anatomical sites that were absent because of previous minor amputation were coded as non-assessable and were not imputed. Pain intensity referred to current lower-limb/foot pain in the remaining limb or anatomical region. Phantom limb pain was not specifically assessed.

### Sample size

As this was an exploratory pilot trial, no formal sample size calculation was performed. The sample was defined by convenience, considering participants availability and adherence to the service. The main objective was to generate preliminary effect estimates and assess protocol feasibility in order to support future confirmatory studies. Therefore, the study was not powered to detect definitive between-group superiority.

### Statistical analysis

Data were analyzed using Statistical Package for the Social Sciences version 26 and complemented by longitudinal modeling in Python. Quantitative variables were described as mean and standard deviation or mean with 95% confidence interval, whereas qualitative variables were described as absolute and relative frequencies. Decimal points were standardized throughout the manuscript.

The distribution of quantitative variables and model residuals was assessed descriptively through visual inspection and normality assessment. Because of the small sample size, repeated-measures structure, and asymmetric distribution of VAS values, nonparametric tests were selected for exploratory longitudinal comparisons. Within-group comparison of neuropathic pain intensity across the five time points was performed using the Friedman test, followed by pairwise post hoc analyses with Bonferroni correction when applicable. Paired comparison of neuropathic symptoms between baseline and the end of the protocol was performed using McNemar test, with Bonferroni adjustment for multiple comparisons.

A linear mixed-effects model was fitted to explore the longitudinal trajectory of neuropathic pain and the interaction between group and time, considering a random intercept for each participant (VAS ~ Group x Time + (1 | Participant)). Fixed effects included group, time, and the group x time interaction. Model fit and assumptions were assessed descriptively using residual inspection. Effect size was expressed using marginal R2, representing the proportion of variance explained by fixed effects, and conditional R2, which included both fixed and random effects.

All randomized participants were analyzed in the groups to which they were allocated. Because all randomized participants completed the follow-up assessments, the intention-to-treat and complete-case analytical samples were identical, and no post-randomization missing outcome data were imputed. A significance level of 5% was adopted for all analyses. Given the pilot design, all analyses were interpreted as exploratory.

### Ethical aspects

The study was approved by the Research Ethics Committee of the university where the research was conducted and was carried out in accordance with the Declaration of Helsinki and Brazilian National Health Council Resolutions 466/12 and 510/16. All participants signed an informed consent form before inclusion in the sample.

## Results

**Fig. 2:**
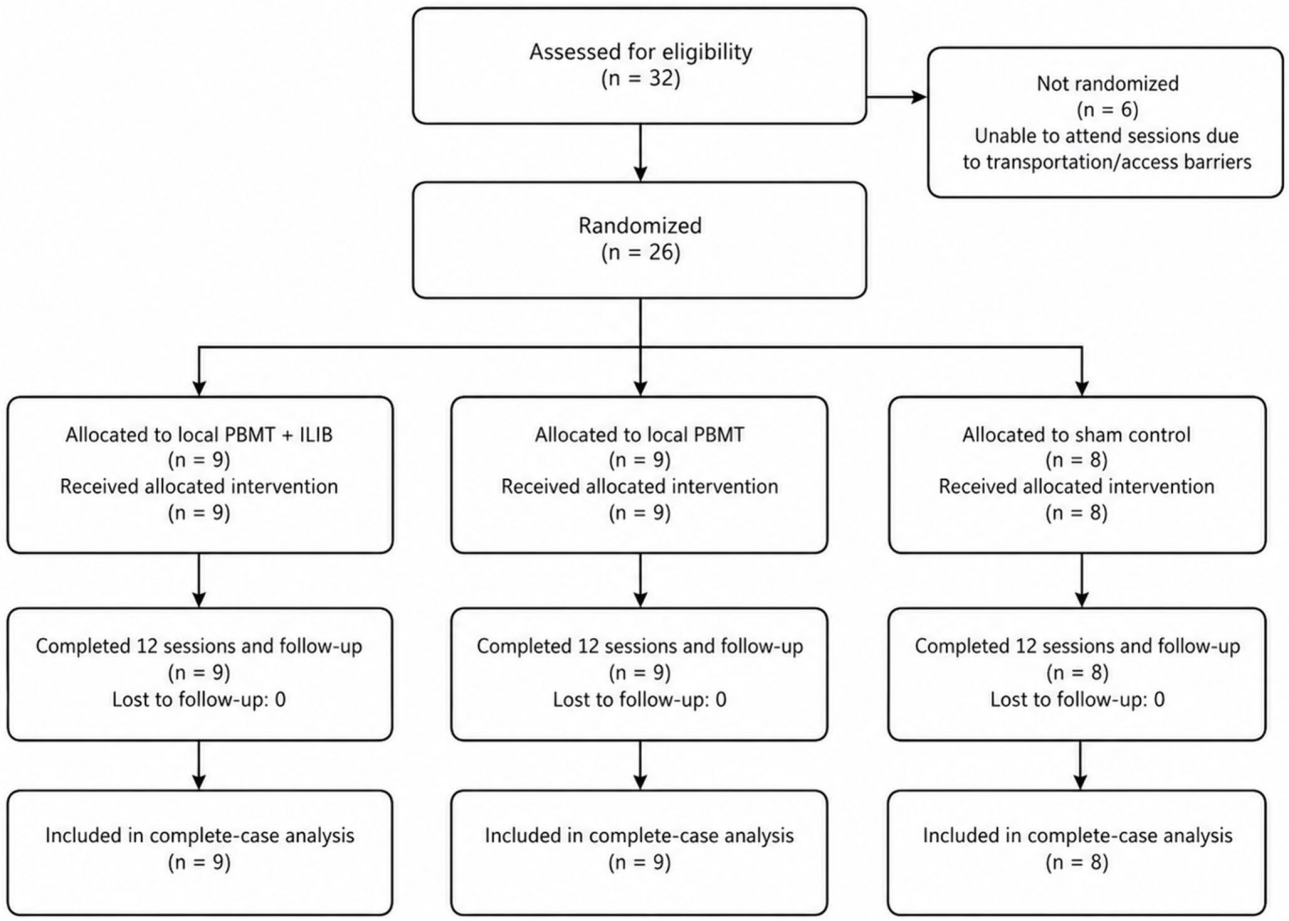
Participant flow diagram. Note: PBMT, photobiomodulation therapy; ILIB, intravascular laser irradiation of blood. Source: authors (2026)

A total of 32 individuals were assessed for eligibility. Six were not randomized because of transportation difficulties and access barriers that prevented attendance at the scheduled intervention sessions. Therefore, 26 individuals with diabetes mellitus were randomized and allocated to three groups: local PBMT + ILIB (*n* = 9), local PBMT (*n* = 9), and sham control (*n* = 8). All randomized participants completed the 12-session protocol and were included in the analysis. Participant flow is presented in Fig. 2.

Overall, neuropathic pain intensity decreased across the five assessment time points, from 3.58 to 0.08 (*p* < 0.001), with significant intragroup reduction in the local PBMT group, in which pain decreased from 4.44 to 0.22 (*p* = 0.007). The local PBMT + ILIB group showed a numerical reduction, from 3.67 to 0.00, although without statistical significance (*p* = 0.093). The sham-control group remained statistically stable (*p* = 0.136). In neuropathy screening, reductions were observed in selected symptoms, especially cramps, from 73.1% to 11.5% (*p* < 0.001), and numbness, from 61.5% to 26.9% (*p* = 0.004).

### Baseline characterization and between-group comparability

The analysis of baseline sociodemographic and clinical characteristics, presented in Table 2, showed no statistically significant differences between groups (*p* > 0.05), indicating acceptable baseline comparability for an exploratory pilot trial. Mean age was similar across groups: local PBMT + ILIB, 57.56 +/- 13.13 years; local PBMT, 57.89 +/- 14.04 years; and sham control, 63.00 +/- 12.93 years (*p* = 0.697). Mean body mass index values were also similar: local PBMT + ILIB, 29.73 +/- 9.66; local PBMT, 28.83 +/- 4.49; and sham control, 30.65 +/- 9.66 (*p* = 0.857).

**Table 2:** Baseline sociodemographic and clinical characteristics of randomized participants

Characteristic	Local PBMT + ILIB (*n* = 9)	Local PBMT (*n* = 9)	Sham control (*n* = 8)	*p*-value
Age, years, mean ± SD	57.56 ± 13.13	57.89 ± 14.04	63.00 ± 12.93	0.697
Male sex, n (%)	5 (55.6)	6 (66.7)	6 (75.0)	0.699
Female sex, n (%)	4 (44.4)	3 (33.3)	2 (25.0)	0.699
BMI, kg/m², mean ± SD	29.73 ± 9.66	28.83 ± 4.49	30.65 ± 9.66	0.857
Baseline moderate-to-severe pain, n/N (%)	4/9 (44.4)	5/9 (55.6)	3/8 (37.5)	-
Diabetes duration > 10 years, n (%)	4 (44.4)	7 (77.8)	2 (25.0)	0.408
History of micro- or macrovascular complications, n (%)	5 (55.6)	6 (66.7)	6 (75.0)	0.414
History of limb ulcers, n (%)	2 (22.2)	5 (55.6)	3 (37.5)	0.347
History of limb amputations, n (%)	2 (22.2)	3 (33.3)	1 (12.5)	0.594
Smoking history, n (%)	5 (55.6)	1 (11.1)	1 (12.5)	0.057
Ambulates without assistance, n (%)	7 (77.8)	8 (88.9)	7 (87.5)	0.779

Values are presented as mean ± standard deviation or n (%). p-values refer to baseline between-group comparisons, where applicable. BMI, body mass index; ILIB, intravascular laser irradiation of blood; PBMT, photobiomodulation therapy; SD, standard deviation; VAS, Visual Analog Scale. Moderate-to-severe pain was defined as VAS ≥ 4 at baseline and is presented descriptively

Source: authors (2026)

### Neuropathic pain trajectory

The overall and group-specific analysis of neuropathic pain intensity using the VAS. The overall baseline mean was 3.58 (95% CI: 2.00 to 5.15), decreasing to 2.12 (95% CI: 0.78 to 3.45) at the second time point, 1.42 (95% CI: 0.34 to 2.50) at the third, 0.65 (95% CI: 0.05 to 1.26) at the fourth, and 0.08 (95% CI: -0.03 to 0.19) at the fifth time point. The Friedman test showed a statistically significant difference across time points (*p* < 0.001).

At baseline, using individual-level VAS scores, 14 participants (53.8%) had no or mild current pain (VAS 0–3), 4 (15.4%) had moderate pain (VAS 4–6), and 8 (30.8%) had severe pain (VAS 7–10). The proportion of participants with moderate-to-severe baseline pain (VAS > = 4) was 4/9 (44.4%) in the local PBMT + ILIB group, 5/9 (55.6%) in the local PBMT group, and 3/8 (37.5%) in the sham-control group. These data describe the pain-intensity spectrum of the randomized cohort, while all randomized participants met the clinical eligibility criterion of symptomatic DPN.

Post hoc comparisons suggested that the largest reduction occurred between baseline and the final assessment; however, because of the exploratory pilot design and multiple comparisons, these results were interpreted descriptively. In the local PBMT group, the mean VAS score decreased from 4.44 (95% CI: 1.37 to 7.52) at the first time point to 0.22 (95% CI: -0.12 to 0.56) at the fifth, with a significant difference over time (*p* = 0.007).

In the local PBMT group, the mean VAS score decreased from 4.44 (95% CI: 1.37 to 7.52) at the first time point to 0.22 (95% CI: -0.12 to 0.56) at the fifth, with a significant difference over time (*p* = 0.007). In the local PBMT + ILIB group, the mean score declined from 3.67 (95% CI: 0.14 to 7.19) to 0.00 at the end of follow-up, although the Friedman test did not indicate statistical significance (*p* = 0.093). In the sham-control group, the mean score decreased from 2.50 (95% CI: -0.11 to 5.11) to 0.00, without statistical significance (*p* = 0.136). These intragroup findings should not be interpreted as evidence of comparative superiority because the group × time interaction in the mixed-effects model was not statistically significant.

### Neuropathy screening and sensory assessment

The paired analysis comparing the initial and final time points of the clinical protocol is presented in Table 3. The frequency of numbness decreased from 61.5% to 26.9% (*p* = 0.004). Stabbing pain in the legs or feet decreased from 57.7% to 26.9% (*p* = 0.008). Cramps decreased from 73.1% to 11.5% (*p* < 0.001). Muscle weakness decreased from 38.5% to 7.7% (*p* = 0.039). Dry skin with cracks decreased from 57.7% to 11.5% (*p* = 0.002). No significant difference was observed for burning pain, worsening of symptoms at night or during the day, or sensory perception while walking.

**Table 3:** Paired comparison of neuropathic symptoms from baseline to final assessment

Neuropathic symptom	Baseline Yes *n* (%)	Final Yes *n* (%)	*p*-value
Numbness in the feet in recent days	16 (61.5)	7 (26.9)	0.004
Burning pain in the legs and/or feet in recent days	14 (53.8)	8 (30.8)	0.146
Stabbing pain in the legs or feet in recent days	15 (57.7)	7 (26.9)	0.008
Cramps in the legs and/or feet in recent days	19 (73.1)	3 (11.5)	< 0.001
Muscle weakness most of the time	10 (38.5)	2 (7.7)	0.039
Skin on the feet dry enough to crack	15 (57.7)	3 (11.5)	0.002
Able to feel the feet while walking	24 (92.3)	26 (100.0)	0.500
Symptoms in the feet worsen at night or during the day	19 (73.1)	14 (53.8)	0.125

Comparisons were performed using the McNemar test. Symptoms were assessed using items from the Michigan Neuropathy Screening Instrument (MNSI) and related clinical screening questions

Source: authors (2026)

In the stratified exploratory analysis, the local PBMT + ILIB group showed reductions in numbness, from 66.7% to 0% (*p* = 0.031), and cramps, from 66.7% to 0% (*p* = 0.031). The local PBMT group showed reductions in cramps, from 88.9% to 22.2% (*p* = 0.031), and dry skin with cracks, from 88.9% to 22.2% (*p* = 0.031). The sham-control group showed no statistically significant differences in the evaluated symptoms (*p* > 0.05). Because of the small sample size and multiple comparisons, these subgroup findings were interpreted as exploratory clinical signals only and are summarized narratively rather than presented as an additional table.

Sensory findings were summarized descriptively because the previous extensive sensory table was long and difficult to interpret in the context of this pilot sample. Thermal alteration in the right foot decreased from 26.9% at the first time point to 3.8% at the fifth. In the left foot, thermal alteration decreased from 69.2% to 42.3%. Sensitivity assessed by monofilament testing remained characterized by a high proportion of absent perception, especially in the right foot, where 73.1% had no perception at the first time point and 80.8% at the fifth. These findings suggest that the observed short-term changes occurred predominantly in subjective symptoms of pain and discomfort, with limited change in protective tactile sensitivity assessed by monofilament testing.

### Longitudinal mixed-effects model

A significant effect of time was observed on the reduction of neuropathic pain (beta = -0.78; 95% CI: -1.24 to -0.31; *p* = 0.001), indicating a mean decrease of 0.78 points on the VAS at each successive time point of the protocol, regardless of group. No statistically significant baseline differences were identified between groups in the model intercept (*p* > 0.05), suggesting acceptable initial balance across allocations.

The group x time interaction did not reach statistical significance (local PBMT x time: beta = -0.32; *p* = 0.337; local PBMT + ILIB x time: beta = 0.14; *p* = 0.685), indicating that, although all groups showed a tendency toward pain reduction, there was no evidence that the slope of improvement formally differed between interventions. Thus, no definitive between-group superiority was demonstrated.

The model showed a marginal R2 of 0.29, indicating that 29% of the variability in pain was explained by the fixed effects, group and time, and a conditional R2 of 0.49, suggesting that 49% of the total variability was explained when individual heterogeneity was taken into account. These values indicate that individual variability contributed meaningfully to the pain trajectory and should be considered when designing larger confirmatory trials. The estimated pain trajectories are described in the mixed-effects model results and summarized in Table 4.

**Table 4:** Linear mixed-effects model for neuropathic pain intensity over time

Fixed effect	β coefficient	SE	95% CI	*p*-value
Intercept	3.53	0.92	1.74 to 5.33	< 0.001
Local PBMT group	2.01	1.30	-0.53 to 4.55	0.120
Local PBMT + ILIB group	-0.40	1.33	-3.01 to 2.22	0.767
Time	-0.78	0.24	-1.24 to -0.31	0.001
Local PBMT group × time	-0.32	0.34	-0.98 to 0.34	0.337
Local PBMT + ILIB group × time	0.14	0.35	-0.54 to 0.82	0.685

Linear mixed-effects model: VAS ~ Group × Time + (1 | Participant). The sham-control group was used as the reference category. Marginal R² = 0.29 and conditional R² = 0.49. CI, confidence interval; ILIB, intravascular laser irradiation of blood; PBMT, photobiomodulation therapy; SE, standard error; VAS, Visual Analog Scale

Source: authors (2026)

Overall, the estimated trajectories indicated a progressive reduction in neuropathic pain intensity over time in all groups. Although the local PBMT group showed a numerically steeper decline than the other groups, the group x time interaction was not statistically significant; therefore, this numerical pattern should be interpreted cautiously and not as evidence of superiority.

## Discussion

In this randomized pilot trial, local PBMT, administered alone or combined with ILIB, was feasible in an outpatient setting and was associated with preliminary short-term reductions in neuropathic pain intensity and selected neuropathic symptoms. The completion of the 12-session protocol among all randomized participants suggests that the intervention schedule was operationally feasible among participants who were able to attend the health service. However, consistent with the role of pilot trials as studies intended primarily to refine procedures and estimate preliminary signals rather than establish definitive efficacy, these findings must be interpreted cautiously because the sample was small and no significant group x time interaction was detected.

The main longitudinal finding was a significant effect of time on VAS pain reduction, indicating that pain intensity decreased during the 23-day protocol regardless of group allocation. Although the local PBMT group showed a statistically significant intragroup reduction and a numerically steeper pain trajectory, the absence of a statistically significant group x time interaction means that comparative superiority between interventions cannot be concluded from this pilot study. This distinction is clinically important in DPN research, because painful symptoms frequently fluctuate over time and may be influenced by attention, expectation, concomitant medication use, and baseline severity [1, 7].

The baseline pain profile also supports a cautious interpretation. The overall mean VAS score of 3.58 indicates mild pain on average, although 46.2% of participants had moderate-to-severe current pain at baseline. DPN is clinically heterogeneous, and patients may present with pain, numbness, paresthesia, sensory loss, autonomic changes, or combinations of these features ([1]; [2]). In the present study, eligibility was based on a clinical presentation compatible with symptomatic DPN, including pain symptoms and sensory complaints, rather than on a rigid VAS severity threshold. This broader symptomatic criterion is aligned with the outpatient pilot setting, but baseline heterogeneity may have influenced the magnitude of change observed across follow-up.

The observed reductions in numbness, cramps, stabbing pain, muscle weakness, and dry skin with fissures suggest possible short-term modulation of symptom domains beyond pain intensity alone. This is clinically relevant because DPN involves sensory, motor, autonomic, and microvascular components [4, 18]. Small-fiber involvement contributes to pain, thermal alterations, and sudomotor dysfunction, whereas large-fiber involvement may affect protective sensation, balance, and mechanical loading [2, 18]. Nevertheless, because these outcomes were exploratory, self-reported or clinically assessed, and based on a small sample, they should be interpreted as preliminary signals rather than definitive evidence of therapeutic efficacy.

The biological plausibility of PBMT in neuropathic conditions has been attributed to mitochondrial photoacceptor activation, modulation of oxidative stress, nitric oxide-mediated vascular responses, and attenuation of inflammatory signaling [8, 19]. These pathways are relevant to DPN because mitochondrial dysfunction, impaired microcirculation, endoneurial hypoxia, and chronic low-grade inflammation contribute to peripheral nerve injury and neuropathic symptoms [5, 18]. In experimental and clinical neuropathic pain contexts, PBMT has been proposed to modulate neuronal excitability, inflammatory chemokine signaling, and tissue repair responses [19, 20]. However, the present study did not include objective neurophysiological or biochemical outcomes, such as nerve conduction studies, quantitative sensory testing, inflammatory markers, or neuronal biomarkers. Therefore, mechanistic interpretations should remain speculative.

The improvement also observed in the sham-control group reinforces the complex and multifactorial nature of chronic neuropathic pain. Painful DPN is influenced not only by peripheral nerve damage, but also by central modulation, emotional factors, sleep quality, expectations, and concurrent pharmacological treatment [1, 7]. Therapeutic expectation, placebo effects, increased clinical attention during follow-up, natural symptom fluctuation, residual confounding due to medication use, and regression to the mean may therefore have contributed to the reductions observed across groups. These factors are particularly important in pilot trials with small samples and self-reported outcomes.

The short duration of follow-up is another important consideration. The protocol lasted 23 days, and outcomes were assessed only during this short intervention period. Therefore, the observed changes may represent transient symptomatic relief rather than sustained improvement in neuropathic status. This limitation is especially relevant because DPN is a chronic progressive condition in which durable clinical benefit should ideally be demonstrated through longer post-treatment follow-up and objective measures of nerve function [6, 18]. Larger trials should include post-treatment follow-up periods to determine whether any clinical benefits persist after the intervention is discontinued.

Previous randomized and controlled studies have reported favorable effects of PBMT-related interventions on neuropathic pain, quality of life, neuronal biomarkers, nerve conduction, or plantar pressure distribution in individuals with DPN. Anju et al., [15] reported improvements in pain, quality of life, and neuron-specific biomarkers after PBMT in individuals with type 2 diabetes and peripheral neuropathy. Korada et al., [16] summarized evidence suggesting potential benefits of PBMT on neuropathic pain, nerve conduction, and plantar pressure distribution, while Amoli et al., [11] reported clinical and quality-of-life changes after PBMT, alone or combined with another physical modality, in individuals with type 2 diabetes. The present pilot study adds to this literature by exploring longitudinal pain trajectory and selected neuropathic symptoms in a three-arm design that included local PBMT, local PBMT combined with ILIB, and sham control. However, because of the limited statistical power and lack of significant between-group interaction, these results should be used primarily to inform the design of future confirmatory trials.

### Limitations and contributions

This study has limitations inherent to its pilot design. The small sample size restricts statistical power to detect comparative differences between interventions and increases susceptibility to random variation. Consequently, effect-size estimates and observed symptom changes should be interpreted as preliminary.

Eligibility was based on the presence of symptomatic DPN, reflecting the clinical diversity encountered in outpatient practice. As a result, baseline pain intensity varied among participants, which may have contributed to differences in the magnitude of symptom change observed during follow-up. Future studies may explore stratification according to baseline pain severity to further investigate potential differences in treatment response across clinical subgroups.

Information on antidiabetic and neuropathic pain medications was obtained as part of routine clinical characterization; however, detailed data regarding medication classes, dosages, and treatment modifications during follow-up were not systematically collected.

The study adopted blinding procedures consistent with the nature of the interventions. Participants and the statistician remained blinded to group allocation. Due to the operational characteristics of the protocol, the therapists responsible for delivering the interventions also conducted the clinical assessments, a factor that should be considered when interpreting the findings.

Because the primary aim of this pilot study was to evaluate feasibility and preliminary clinical outcomes, objective neurophysiological assessments and biomarkers were not included. Future studies may incorporate complementary measures, such as nerve conduction studies, quantitative sensory testing, autonomic function assessments, and biomarkers related to neural function, to further explore the physiological effects and potential mechanisms associated with these interventions.

Despite these limitations, this study contributes by providing feasibility data and preliminary longitudinal information on neuropathic pain trajectories in an outpatient setting. In addition, it explores selected sensory, motor, and autonomic symptom domains while comparing local PBMT, local PBMT combined with ILIB, and sham control within a single pilot design. These findings may inform the design of larger randomized controlled trials with adequate statistical power, longer follow-up periods, and incorporation of complementary objective outcomes.

## Conclusion

This randomized pilot study suggests that local PBMT, administered alone or combined with ILIB, was feasible in an outpatient setting for individuals with DPN. Preliminary exploratory findings indicated short-term reductions in neuropathic pain intensity and selected neuropathic symptoms, particularly numbness and cramps. However, sensory responses showed limited change, especially protective tactile sensitivity assessed by monofilament testing. No definitive between-group superiority was demonstrated.

These findings should be interpreted cautiously considering the pilot design, small sample size, limited statistical power, heterogeneity in baseline pain intensity, short follow-up period, absence of blinded outcome assessment, lack of objective neurophysiological outcomes, potential placebo effects, residual confounding, and exploratory analysis. Larger, adequately powered randomized controlled trials with rigorous allocation concealment, blinded outcome assessment, standardized dosimetry, longer follow-up, sham-controlled conditions, stratification by baseline clinical characteristics, and objective neurophysiological measures are warranted.

## Data Availability

The data supporting the findings of this study are not publicly available due to privacy and ethical restrictions involving human participants. Data may be made available from the corresponding author upon reasonable request and subject to approval by the relevant ethics requirements.

## Abbreviations

CI: confidence interval
DM: diabetes mellitus
DPN: diabetic peripheral neuropathy
ILIB: intravascular laser irradiation of blood
MNSI: Michigan Neuropathy Screening Instrument
PBMT: photobiomodulation therapy
VAS: Visual Analog Scale

## Declarations

### Clinical trial registration

This study was registered in the Brazilian Registry of Clinical Trials before the beginning of data collection. The registration number is ReBEC: U1111-1297-5743.
